# Modulation of Induced Cytotoxicity of Doxorubicin by Using Apoferritin and Liposomal Cages

**DOI:** 10.3390/ijms151222960

**Published:** 2014-12-11

**Authors:** Jaromir Gumulec, Michaela Fojtu, Martina Raudenska, Marketa Sztalmachova, Anna Skotakova, Jana Vlachova, Sylvie Skalickova, Lukas Nejdl, Pavel Kopel, Lucia Knopfova, Vojtech Adam, Rene Kizek, Marie Stiborova, Petr Babula, Michal Masarik

**Affiliations:** 1 Department of Pathological Physiology, Faculty of Medicine, Masaryk University, Kamenice 5, CZ-62500 Brno, Czech Republic; E-Mails: j.gumulec@gmail.com (J.G.); michaelafojtu@gmail.com (M.F.); 42700@mail.muni.cz (M.R.); sztalm@med.muni.cz (M.S.); 424299@mail.muni.cz (A.S.); 2 Central European Institute of Technology, Brno University of Technology, Technicka 3058/10, CZ-61600 Brno, Czech Republic; E-Mails: vlachova.jana@centrum.cz (J.V.); sylvie.skalickova@gmail.com (S.S.); lukasnejdl@gmail.com (L.N.); paulko@centrum.cz (P.K.); vojtech.adam@mendelu.cz (V.A.); kizek@sci.muni.cz (R.K.); 3 Department of Physiology, Faculty of Medicine, Masaryk University, Kamenice 5, CZ-62500 Brno, Czech Republic; E-Mail: babula@med.muni.cz; 4 Department of Chemistry and Biochemistry, Mendel University in Brno, Zemedelska 1, CZ-61300 Brno, Czech Republic; 5 Department of Experimental Biology, Faculty of Science, Masaryk University, Kamenice 5, CZ-62500 Brno, Czech Republic; E-Mail: l.knopfova@seznam.cz; 6 Department of Biochemistry, Faculty of Science, Charles University, Albertov 2030, CZ-12840 Prague 2, Czech Republic; E-Mail: stiborov@natur.cuni.cz

**Keywords:** doxorubicin, liposome, apoferritin, cancer, cardiotoxicity, modification, encapsulation

## Abstract

Doxorubicin is an effective chemotherapeutic drug, however, its toxicity is a significant limitation in therapy. Encapsulation of doxorubicin inside liposomes or ferritin cages decreases cardiotoxicity while maintaining anticancer potency. We synthesized novel apoferritin- and liposome-encapsulated forms of doxorubicin (“Apodox” and “lip-8-dox”) and compared its toxicity with doxorubicin and Myocet on prostate cell lines. Three different prostatic cell lines PNT1A, 22Rv1, and LNCaP were chosen. The toxicity of the modified doxorubicin forms was compared to conventional doxorubicin using the MTT assay, real-time cell impedance-based cell growth method (RTCA), and flow cytometry. The efficiency of doxorubicin entrapment was 56% in apoferritin cages and 42% in the liposome carrier. The accuracy of the RTCA system was verified by flow-cytometric analysis of cell viability. The doxorubicin half maximal inhibition concentrations (IC_50_) were determined as 170.5, 234.0, and 169.0 nM for PNT1A, 22Rv1, and LNCaP, respectively by RTCA. Lip8-dox is less toxic on the non-tumor cell line PNT1A compared to doxorubicin, while still maintaining the toxicity to tumorous cell lines similar to doxorubicin or epirubicin (IC_50_ = 2076.7 nM for PNT1A *vs.* 935.3 and 729.0 nM for 22Rv1 and LNCaP). Apodox IC_50_ was determined as follows: 603.1, 1344.2, and 931.2 nM for PNT1A, 22Rv1, and LNCaP.

## 1. Introduction

The anthracycline antibiotic family comprises hundreds of analogues, but only a few are in actual clinical use. The best known and the most widely used are doxorubicin and epirubicin.

Doxorubicin is an effective chemotherapeutic drug in a wide range of cancers, including both hematological and solid tumors [1]. The therapeutic activity of doxorubicin is achieved through the processes of intercalating into DNA, inhibiting topoisomerase II, and preventing DNA and RNA synthesis [2].

One of the biggest hurdles encountered in cancer therapy includes the problem of dose-dependent toxicity toward the non-cancerous cells because high doses of drug treatment were generally required owing to poor drug accessibility to the tumor site. The total lifetime dose of the doxorubicin is limited to 550 mg/m^2^. As the lifetime accumulative dose approaches 500 mg/m^2^ and beyond, life-threatening cardiomyopathy becomes more likely, which can lead to dilated cardiomyopathy (DCM) and congestive heart failure (CHF) in up to 20% of cases [3]. It has also been shown that doxorubicin can produce acute toxicity due to bone marrow attenuation, alopecia and oral ulceration [4].

The newer anthracyclines, such as epirubicin, and idarubicin have higher lipophilicity and putatively greater safety. However, the risk of inducing cardiomyopathy is not abated; the median cumulative dose of epirubicin to the development of symptomatic CHF is 1134 mg/m^2^ [5]. Early studies found that encapsulation of doxorubicin inside liposomes decreases the cardiotoxicity associated with the free form of the drug, while maintaining anticancer potency [6,7,8,9,10,11,12,13]. Since the liposomal form of the drug is less toxic for the nondividing cells, increased drug dosages can be administered, ensuing in improved efficacy and an increase in the therapeutic index. Furthermore, Rahman *et al.* have shown that liposome-encapsulated doxorubicin modulates the multidrug-resistance (MDR) phenotype in cancer cells by changing the function of *p*-glycoprotein. The liposomes bound to *p*-glycoprotein [14,15], which was associated with enhanced cellular drug accumulation and altered drug distribution inside the cells [14,16,17,18]. The modulation of MDR phenotype by liposomes has been also demonstrated in mice transfected with a functional human *mdr-l* gene [19].

Another promising delivery system for anticancer drugs seems to be apoferritin [20]. Apoferritin is the iron-free form of ferritin, a naturally-occurring iron-storage protein consisting of 24 protein subunits. Its protein subunits assemble to form a hollow cage into which diverse substances, such as drugs, can be placed. Furthermore, ferritin is internalized by some tumors, which can enable targeting to those tumorous tissues [20]. Using apoferritin as a nanocarrier has the potential to move undetected through the body without inducing any resistance from the immune system of the patient.

An invention of a drug delivery system, which is biocompatible, stable, and non-toxic for healthy tissue, but still has high anticancer potency, is a great challenge in anticancer drug research. We synthetized novel apoferritin- and liposome-coated forms of doxorubicin (“Apodox” and “Liposome-8”) and compared their toxicity with doxorubicin and Myocet on prostate cell lines. Three different prostatic cell lines PNT1A, 22Rv1, and LNCaP were chosen as a model of prostate cancer.

Cell line 22Rv1 is derived from a primary tumor and LNCaP represents secondary tumor derived from lymph node metastasis. PNT1A cells are androgen-receptor positive and express wild-type p53. This line was used as a representative of healthy prostatic tissue, taking into account a limitation resulting from faster proliferative rate of PNT1A compared with low basal proliferative rate in normal prostatic glands [21].

The point of interest of this study was to compare the toxicity of the modified doxorubicin forms with commercially available forms using MTT (3-[4,5-dimethylthiazol-2-yl]-2,5-diphenyltetrazolium bromide) assay, real-time cell impedance-based cell growth method, and flow cytometry.

## 2. Results

### 2.1. Characteristics of Modified Doxorubicin Forms

**Figure 1:**
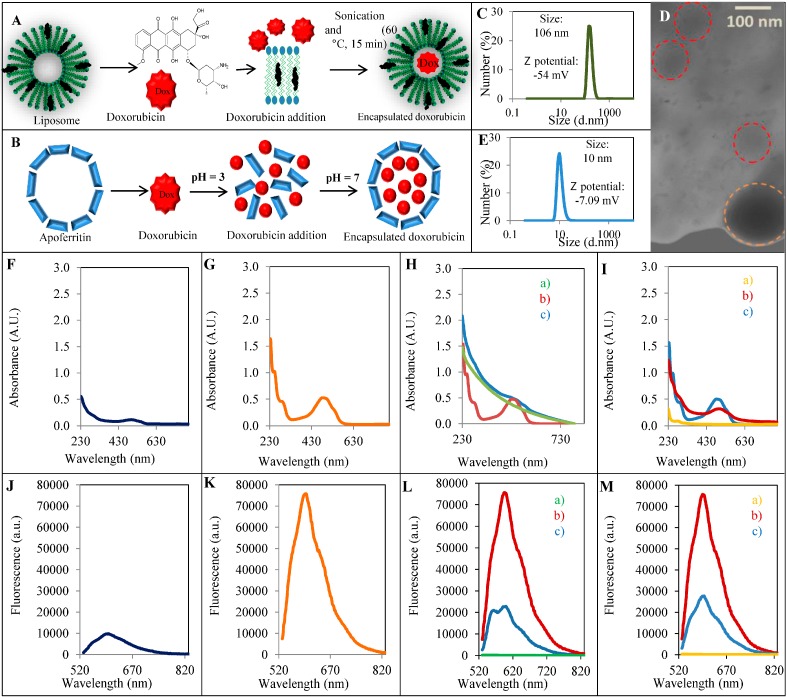
Scheme of encapsulation of doxorubicin into (**A**) the liposome modified by cholesterol and (**B**) apoferritin. (**C**) Size distribution of liposome and (**D**) microphotography of liposomes. Liposomes are highlighted with a circle (red circle = 100 nm and orange circle = 200 nm); (**E**) Size distribution of apoferritin. Absorbtion spectra in the range 230–800 nm of (**F**) 100 µg/mL Myocet; (**G**) Epirubicin; (**H**) Liposome (a), Doxorubicin (b), Encapsulated Doxorubicin into the liposome (c); (**I**) Apoferritin (a), Doxorubicin (b), Encapsulated Doxorubicin into the apoferritin (c); Emission spectra in the range 520–820 nm of (**J**) 100 µg/mL Myocet; (**K**) Epirubicin; (**L**) Liposome (a), Doxorubicin (b), Encapsulated Doxorubicin into the liposome (c); (**M**) Apoferritin (a), Doxorubicin (b), Encapsulated Doxorubicin into the apoferritin (c). The excitation wavelength was 510 nm.

First, we prepared encapsulated 100 µg/mL doxorubicin into 1 mg/mL liposome and 1 mg/mL of apoferritin as is shown in Figure 1A,B. Details of liposome and apoferritin preparation as well as doxorubicin encapsulation are described in caption 4.2 and 4.3. The size and zeta potential of the nanovesicles were measured using a zetasizer. According to the results, the light scattering measurement of liposome (Figure 1C) showed that the median size is 106 nm (40–250 nm) with a small population of liposomes of average size about 800 nm. To prove stability of liposomes, the measurement of the zeta potential (or charge density) was carried out. The high value of zeta potential (−54 ± 0.15 mV) shows high stability as a result of its permanent negative charges on the surface of liposomes. On the Figure 1D is microphotograph of used liposome (highlighted in circle). Subsequently, we performed a light scattering measurement of apoferritin (Figure 1E). Our results proved that the median size of particles is 10 nm (5–15 nm) which is in a good agreement with other publications [1]. High stability of apoferritin and its negative charge a results in a high value of zeta potential (−7.09 mV).

Spectrophotometric characterization of epirubicin, myocet, and doxorubicin encapsulated into the liposome was carried out by measuring the absorbance spectra of studied variants of doxorubicin in the range from 230 to 800 nm of studied variants of doxorubicin. Figure 1E shows the absorbance maximum 0.1 absorbance units (AU) of (100 µg/mL) myocet at 496 nm. Epirubicin shows higher absorption maximum 0.52 AU at 490 nm in comparison with myocet (Figure 1F). Subsequently, we compared the absorbance spectra of Liposome (a), Doxorubicin (b), and encapsulated Doxorubicin into the liposome (c). The results indicate that the liposomes do not show the highest absorbance. Absorbance of doxorubicin 0.49 AU at 490 m is comparable with epirubicin. Absorbance of encapsulated doxorubicin into the liposome shows a decreasing trend with a small peak at 590 nm and with an absorbance maximum of 0.23 AU (Figure 1G). In addition, from the graph in Figure 1H, the low absorbance of apoferritin is evident. In the case of encapsulated doxorubicin into apoferritin, the absorbance maximum of 0.23 AU at 590 nm is higher in comparison with doxorubicin encapsulated into liposome.

Subsequently, the fluorescence spectra of anticancer drugs in the range 520–820 nm at λ = 510 nm of anticancer drugs were measured. Myocet (Figure 1I) shows the fluorescence maximum of 9654 AU at 596 nm, while this value for epirubicin is 75,675 AU at 596 nm, representing an 87% decrease of fluorescence caused by encapsulation of anticancer drug into the liposome. The same trend was estimated for doxorubicin and liposomal doxorubicin. The liposome alone does not have any fluorescence properties, after encapsulation of doxorubicin there is an increase of fluorescence to 22,686 AU at 596 nm what is in comparison with doxorubicin alone enhancement by 70%. In the case of apoferritin encapsulated doxorubicin (27,601 AU at 596 nm), the absorbance is decreaseing by 63% in comparison with doxorubicin and apoferritin showing no fluorescence. From obtained results we estimated the total 72.4 µM (42 µg/mL) doxorubicin was encapsulated into the total amount of 20 mg of liposome carriers. Consequently, cholesterol content was quantified in liposomal particles. Total 0.1 µg/mL was determined. Thus, the prepared liposomes contained 210 µg of doxorubicin per micromole of cholesterol. With regard to apodox, 56 µg/mL of doxorubicin (96.5 µM) was encapsulated into apoferritin cages. Final concentration of apoferritin was 1 mg/mL. Initial concentration of doxorubicin was 100 µg/mL (172.4 µM) in both cases. It follows that the efficiency of doxorubicin entrapment was 42% in case of liposome carrier and 56% in case of apoferritin.

### 2.2. Stability of Nanocarriers

**Figure 2:**
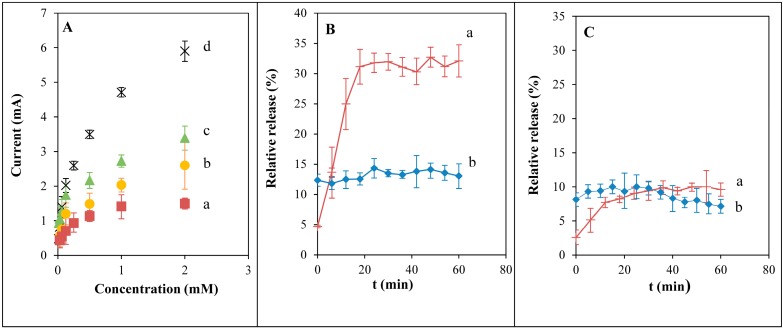
(**A**) Calibration curves of doxorubicine determined in phosphate buffer pH 7.4 with various content of serum as follows: (a) 0%, (b) 5%, (c) 7.5%, and (d) 10%; (**B**) Current signals of the released doxorubicin (related to the enclosed concentration) from liposome (a) and apoferritin (b) in phosphate buffer at pH 7.4 over a 60-min period; (**C**) Current signals of the released doxorubicin from liposome (a) and apoferritin (b) in 5% serum phosphate buffer at pH 7.4 over a 60-min period. Displayed as a mean ± S.E.

We determined doxorubicin in concentration range from 0 to 2 mM in solutions of various concentration of serum (in PBS), Figure 2A. We choose 5% serum phosphate buffer as a model condition for the following experiment, because of sufficient sensitivity for such determinations. Next we tested the stability of complex doxorubicin-liposome and doxorubicin-apoferritin in phosphate buffer pH 7.4 (Figure 2B). Here the difference of releasing amount was obvious for both carriers. Liposome seems to be more sensitive to the phosphate buffer pH 7.4. Apoferritin is stable in the time period from 0 to 60 min. Effect of serum presence was tested in time interval from 0 to 60 min (Figure 2C). Here the decrease of signal observed is related to the determinations in PBS only. Change in relative releasing amount in apoferritin was about 2% and in liposome about 8%. Both carriers seem to be relatively stable in the phosphate buffer pH 7.4 with 5% serum in the time period from 0 to 60 min. Liposome structure was more sensitive to the presence of 5% serum phosphate buffer at pH 7.4.

### 2.3. Comparison of Toxicity of Modified Doxorubicin

First, toxicity of the modified forms of doxorubicin was analyzed. Factorial analysis of variance (ANOVA) was conducted to compare the effect of modified forms of doxorubicin on prostatic cell lines, all other factors were adjusted. There was a significant effect of the form of doxorubicin used on cell lines *F*(4, 68) = 20.49, *p* < 0.0001. *Post-hoc* comparisons using Bonferroni test indicated the highest toxicity of the epirubicin and doxorubicin, with mean half-maximal lethal dose (inhibition concentrations, IC_50_) 118.0 and 268.9 nM, respectively. Toxicity of Apodox and Lip-8-dox was significantly lower; IC_50_ = 1095.0 and 1014.5 nM, respectively. Myocet showed the lowest toxicity with mean IC_50_ = 1915.4 nM (Table 1).

Consequently, the effect of cell lines was analyzed in order to assess, if the sensitivity of individual cell lines to cytostatics differs significantly. After adjustment to all other variables, there was no significant effect of cell line, *F*(2, 68) = 0.46, *p* = 0.62 (Table 1). Despite the statistical insignificance, the highest sensitivity to cytostatics was observed in 22Rv1 cell line and the lowest sensitivity was observed in non-tumor PNT1A cell line.

To reveal, whether this trend in cell lines is common for all cytostatics, a combined effect of cytostatics and cell lines was analyzed. Using this test, significant differences were observed, *F*(8, 68) = 3.08, mp = 0.005. However, the only significant trend between combined effect of cytostatics and cell lines was determined in Myocet; sensitivity of LNCaP cell line to this cytostatic was significantly lower compared to the non-tumor PNT1A cell line (IC_50_ = 3216.1 and 1259.9 nM, respectively). Results demonstrated in previous paragraphs did not take into concern type of viability assay. To check whether these assays provide identical results, MTT and real-time cell impedance-based cell growth method (RTCA) IC_50_ values were analyzed after adjustment of all other variables. Impedance-based RTCA IC_50_ values are on average 1.6-fold lower compared to metabolic-based MTT assay, *F*(1, 68) = 6.48, *p* = 0.013, (Table 1).

**Table 1:** Comparison of half-maximal inhibition concentrations (IC_50_) for particular cell lines, cytostatics and used assay. S.E., standard error. Differences were considered significant, when *p* < 0.05. * Significantly different from doxorubicin (Analysis of variance (ANOVA) followed by Bonferroni *post hoc* testing) † Significantly different from epirubicin (ANOVA followed by Bonferroni *post hoc* testing) ‡ Significantly different from lip-8-dox (ANOVA followed by Bonferroni *post hoc* testing) § Significantly different from apodox (ANOVA followed by Bonferroni *post hoc* testing) ‖ Significantly different from myocet (ANOVA followed by Bonferroni *post hoc* testing) $ Significantly different from MTT (ANOVA followed by Bonferroni *post hoc* testing).

Factor	Level of Factor	*N*	IC_50_ (Mean ± S.E.)	Significant Difference (At *p* < 0.05)
Cytostatic *F*(4, 68) = 20.49, *p* < 0.0001	Epirubicin	21	118.0 ± 27.2	‡, §, ‖
	Doxorubicin	18	268.9 ± 43.6	§, ‖
	Apodox	22	1095.0 ± 163.8	*, †, ‖
	Lip-8	16	1014.5 ± 209.8	†, ‖
	Myocet	21	1915.4 ± 417.3	*, †, ‡, §
Cell line *F*(2, 68) = 0.46, *p* = 0.47	PNT1A	36	936.3 ± 167.9	
	22RV1	32	834.5 ± 161.5	
	LNCaP	30	915.1 ± 299.6	
Method *F*(1, 68) = 6.48, *p* < 0.013	MTT	31	1200.1 ± 316.1	
	RTCA	67	756.1 ± 98.1	$

Taken together, these results suggest that doxorubicin modified by a liposome or apoferritin is less toxic to prostatic cell lines. The commercially available liposomal modification Myocet exhibited the lowest cytotoxicity among all the studied antineoplastic agents; it also showed a less toxic effect on LNCaP derived from the secondary site compared to non-tumor PNT1A. However, differences between assays were revealed, therefore, the type of the assay has to be taken into account when analysing the cytotoxic effects of individual doxorubicin modifications on individual cell lines. Therefore, detailed results of these assays were analysed separately by the assay type in the next step.

#### Comparison of MTT and Real-Time Cell Impedance-Based Cell Growth Method (RTCA) Viability Assays

In this step, results obtained only by MTT were analyzed. Results are not in agreement with previous overall results, that did not take into account viability assay, since these data do not show significant effect of cytostatics and cell lines on cell toxicity, *F*(8, 16) = 1.32, *p* = 0.30.

Further, results obtained by RTCA were analyzed. The combined effect of cytostatics and cell line affected inhibition concentrations significantly; *F*(8, 52) = 2.24, *p* = 0.039. In contrast to MTT and overall results, RTCA-based results show that the Lip-8-modified form of doxorubicin is less toxic on the non-tumor cell line PNT1A compared to doxorubicin, while still maintaining the toxicity to tumorous cell lines (Table 2) (IC_50_ = 2076.7 nM for PNT1A *vs.* 935.3 and 729.0 nM for 22Rv1 and LNCaP).

To identify the relations between MTT and RTCA, Spearman correlation was calculated on mean IC_50_ values. Significant, but relatively weak positive correlation was observed (rSp = 0.48, *p* = 0.03).

Taken together, since there is only the moderate correlation between methods used, the partial disagreement between the results of ANOVAs needs further elucidation. Therefore, the accuracy of the RTCA-based viability analysis was verified using another method, flow-cytometry.

### 2.4. Flow-Cytometric Viability Analysis

**Figure 3:**
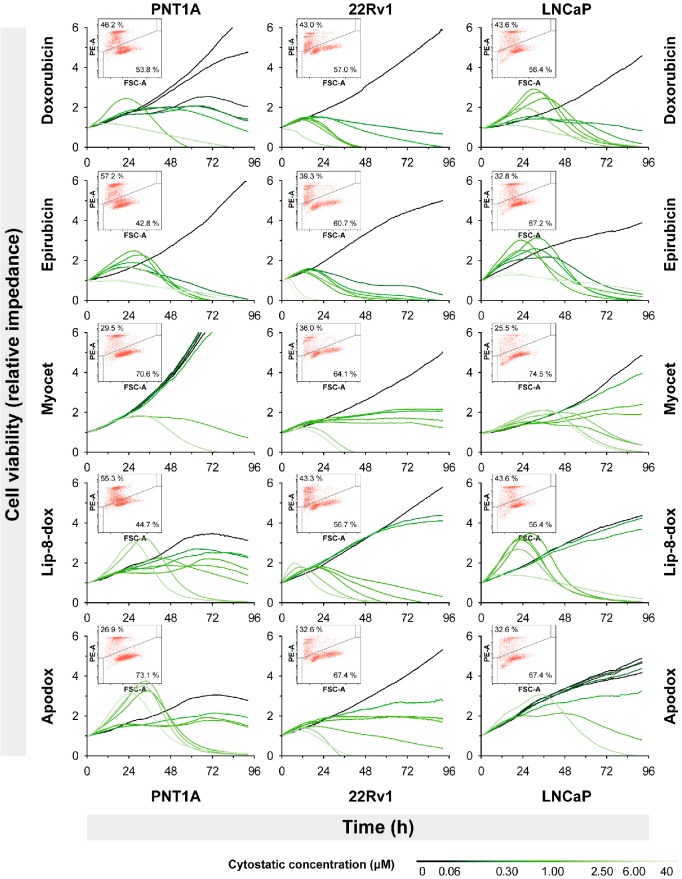
Analysis of prostatic cell line growth treated with modified forms of doxorubicin using RTCA xCELLigence and determination of viable cell numbers by propidium iodide exclusion (inset). Cell lines shown in columns, modified doxorubicin forms in rows. Cell growth expressed as “cell index” on *y*-axis is based on impedance measurement. Increasing concentrations of antineoplastic agents in range 0–40 µM are depicted by green color gradient. Inset: viable cell numbers analyzed after treatment with IC_50_ concentrations of individual cytostatics on individual cell lines as determined by RTCA. FSC-A, forward scatter. Grey line indicate gating threshold for propidium iodide (PI)-positive (upper–left) and -negative cells (lower–right). Percentages in subsequent gating regions indicate number of PI-positive and -negative cells.

**Figure 4:**
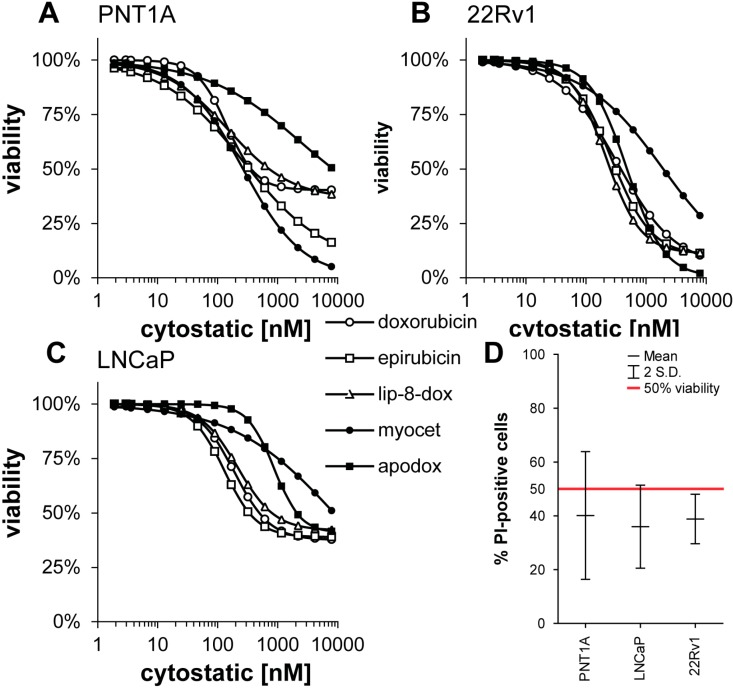
Determination of viability of prostatic cell lines treated with modified forms of doxorubicin using MTT assay (**A**–**C**); (**D**) verification of real-time cell impedance-based cell growth method (RTCA)-determined IC_50_ value. Red line indicates theoretical 50% viability. No significant difference was observed using one sample *t*-test.

To assess, whether the RTCA system determines viability exactly, flow-cytometric analysis of cell viability (% propidium-iodide-positive, *i.e.*, dead) was employed (Figure 3). Cell lines were treated with concentrations of cytostatics defined as IC_50_ by RTCA. Concentrations are shown on Table 2. After the treatment, the portion of propidium-iodide-positive cells was determined as follows: 40.14% ± 11.86%, 38.83% ± 4.61%, and 35.97% ± 7.71% for PNT1A, 22Rv1, and LNCaP, respectively. These values were compared against a theoretical value of 50% using one sample *t*-test. None differed significantly, suggesting RTCA analysis accurately estimated the portion of necrotic cells (Figure 4D).

**Table 2:** Comparison of RTCA-based half-maximal concentrations (IC_50_). S.E., standard error. Differences were considered significant, when *p* < 0.05; * Significantly different from lip-8-dox PNT1A (ANOVA followed by Bonferroni *post hoc* testing); † Significantly different from myocet 22Rv1 (ANOVA followed by Bonferroni *post hoc* testing).

Factor	Cytostatic	Cell Line	*N*	IC_50_ (Mean ± S.E.)	Significant Difference (At *p* < 0.05)
*F*(8, 52) = 2.24, *p* < 0.039	epirubicin	PNT1A	3	12.2 ± 3.5	*
		22RV1	6	49.2 ± 10.5	*, †
		LNCaP	6	107.9 ± 57.0	*, †
	doxorubicin	PNT1A	5	170.5 ± 40.0	*, †
		22RV1	3	234.0 ± 82.1	*
		LNCaP	3	169.0 ± 32.9	*
	apodox	PNT1A	6	603.1 ± 256.8	*
		22RV1	5	1344.2 ± 342.4	
		LNCaP	5	931.2 ± 274.4	
	Lip-8	PNT1A	4	2076.7 ± 353.6	
		22RV1	3	935.3 ± 113.5	
		LNCaP	3	729.0 ± 44.1	
	myocet	PNT1A	8	1047.1 ± 279.5	
		22RV1	4	1657.1 ± 662.9	
		LNCaP	3	1440.0 ± 164.6	

## 3. Discussion

The aim of this study was to encapsulate doxorubicin into apoferritin and liposomal structures and to compare cytotoxic effects caused by newly prepared compounds with commercial ones. 56 µg/mL of doxorubicin (96.5 µM) was encapsulated into the apoferritin cages. The pH gradient method was used to encapsulate higher amount of doxorubicin in lip-8 because the aim was to prepare encapsulated doxorubicin in the same simple manner as in the case of commercially produced Myocet. Efficiency of doxorubicin entrapment was found to be 56%. Our reached amount of encapsulated doxorubicin was thus 19-times higher compared to Simsek *et al.* [22]. Since apoferritin is stable at the physiological pH of 7.4, we suppose that it should not precipitate in blood [23].

Liposomal drug delivery systems have recently been greatly investigated to increase the therapeutic index of chemotherapy [24]. In our study, a total of 72.4 µM (42 µg/mL) doxorubicin was encapsulated into the liposome carrier. Efficiency of doxorubicin entrapment was found to be 42%. In experiments of Gokhale *et al.*, 90% encapsulation of doxorubicin was observed [15]. Nevertheless, an average IC_50_ value of 0.27 µM was determined for free doxorubicin in our studied cell cultures. Considering the efficiency of encapsulation of 72.4 µM, our encapsulation system can likely deliver effective drug amounts for therapeutic applications. Liposomes prepared by us contained 210 µg of doxorubicin per micromole of cholesterol. Tseng *et al.* and Hong *et al.* achieved concentrations from 110 to 130 µg of doxorubicin per micromole of phospholipid [25,26].

Cytotoxicities of doxorubicin, epirubicin, apodox (apoferritin filled with doxorubicin), and of two liposomal forms of doxorubicin (newly prepared liposome-8 and commercial Myocet) on prostate cell lines was compared by using MTT assay and the RTCA system (impedance-based method xCELLigence). As demonstrated in our previous studies [27], precise determination of half-maximal lethal doses of cytostatics is arduous. Results of assays vary among themselves because methods are based on different mechanisms. However, it is not possible to rely solely on the results of one method. The main principle of MTT assay is tetrazolium salts’ reduction to formazan by mitochondrial succinate dehydrogenase (SDH), which is quantified photometrically. Nevertheless SDH is only active in cells with an intact metabolism and respiratory chain. Fasting and oxidative stress, which is present by anthracycline treatment, was associated with a significant decrease of SDH activity, and the reduction was proportional with the decrease in the amount of SDH total protein [28,29]. Therefore, a false reduced viability of MTT assay could be caused by low metabolic activity of cells due to autophagy or senescence [30], which are not rare in cytostatic-treated cells. On the other hand, a false reduced or increased viability can be also caused by changes in cell size and/or adhesion as determined by RTCA. RTCA is based on changes in impedance influenced by cell number, size and adherence thus reference methods for accurate cell viability assessment are necessary. The discrepancy between methods is well illustrated by relatively low correlation power, as determined by Spearman’s rank correlation in this study. In this study, the accuracy of the IC_50_ determination by these methods was verified by flow cytometry (FCM). FCM-determined percentage of propidium-iodide-positive cells correlated with RTCA, suggesting that RTCA reflects cellular viability more accurately and that is not biased by metabolic status, therefore is more suitable for studies on antineoplastic agents.

The cytotoxicity determination is in agreement with studies by Graeser *et al.*, showing doxorubicin IC_50_ values 140 nM for LNCaP [31]. As presumed, doxorubicin and epirubicin are significantly the most toxic forms among studied cytostatics. Commercially available doxorubicin forms and those modified in our lab are of significantly lower toxicity. Although the toxicity of Myocet is the lowest among all of the studied cytostatics, the sensitivity of individual cell types differs significantly. While higher toxicity was observed on cell lines derived from non-tumour tissue (PNT1A) and primary tumor (22Rv1), the toxic effect on cells derived from metastatic sites (LNCaP) was significantly lower in this commercially available modification. These findings indicate a limited efficiency of Myocet in the management of advanced disease.

In contrast, the sensitivity of cell lines to lip-8-dox did show the opposite pattern: although below the level of statistical significance, the lowest toxicity was determined for non-tumour PNT1A, toxicity to tumorous cell lines was distinctly higher, while the highest toxic effect was observed on a cell line derived from secondary tumor—LNCaP. These findings indicate that lip-8-dox is a potential drug for the management of advanced cancer. Nevertheless, faster proliferative rate of PNT1A cell line compared with a low basal proliferative rate in normal prostatic glands should be taken into account [21]. Therefore, we assume that a slowly proliferating non-tumor tissue will be even more resistant compared to the cell line model used in this study. The toxicity of the apoferritin-coated doxorubicin synthetized in our lab, apodox, was 5-fold lower compared to conventional doxorubicin with no significant difference in sensitivity between cell lines.

Inasmuch as liposome-8 and apodox are less toxic compounds with higher IC_50_ value, it seems possible that different mechanisms could be activated by high and low concentrations of tested compounds. It is consistent with the study by Eom *et al.*, who found that chronic exposure of hepatoma cells to high doses of doxorubicin induced apoptosis, whereas lower doses induced senescence and late death by mitotic catastrophe [32]. It was also demonstrated that low doses of doxorubicin are able to induce senescence or autophagy in various types of cells [33,34].

## 4. Experimental Section

### 4.1. Chemical and Biochemical Reagents

Apoferritin from equine spleen, cholesterol, 1,2-dioleoyl-sn-glycero-3-phospho-rac-(1-glycerol) sodium salt, HCl, NaOH and water were purchased from Sigma-Aldrich (St. Louis, MO, USA) in American chemical society purity. Hydrogenated phosphatidylcholine from soybean was a gift from Lipoid GMBH (Ludwigshafen, Germany). Doxorubicin solution 2 mg/mL was obtained from Teva Pharmaceuticals (Opava, Czech Republic). To pipette volumes down to micro and nanolitres, pipettes used were purchased from Eppendorf Research (Eppendorf, Germany) with the highest certified deviation (±12%). The deionized water was prepared using reverse osmosis equipment Aqual 25 (Brno, Czech Republic). The deionized water was further purified by using apparatus MiliQ Direct QUV equipped with the UV lamp (Millipore, Billerica, MA, USA). The resistance was 18 MΩ. The pH was measured using pH meter WTW inoLab (Weilheim, Germany).

### 4.2. Preparation of Apoferritin Filled with Doxorubicin

Apoferritin solution 20 µL (50 mg/mL, equine spleen, Sigma-Aldrich) was diluted with 200 µL of ACS water. Doxorubicin (100 µL, 2 mg/mL) was added and mixture was shaken. 1 M HCl (2 µL) was added and turbidity was observed. Fifteen minutes later 1 M NaOH (2.5 µL) was added and turbidity disappeared. Solution was subsequently shaken on Vortex Genie2 for 2 h (Scientific Industries, Bohemia, NY, USA). Dialyses for 24 h were performed on membrane filter (0.025 µm VSWP, Millipore) against 2 L of water. Thus obtained solution was diluted with ACS water to final volume of 1 mL. This form is designated as “Apodox”.

### 4.3. Preparation of Liposome-8-doxorubicin

Cholesterol (100 mg), 1,2-dioleoyl-sn-glycero-3-phospho-rac-(1-glycerol) sodium salt (100 mg) and phosphatidylcholine (100 mg) were dissolved in chloroform (4.5 mL). A lipid film was obtained by rotary evaporation of solvent and residual chloroform was blown out by nitrogen.

Doxorubicin solution (1 mL, 4 mg/mL) (pH = 7) was added to liposome (20 mg) and shaken for 3 h on Vortex. Sample was homogenized with Ultra-Turrax T8 (IKA Werke GmbH, Staufen, Germany) for 3 min. The mixture was then heated and shaken for 10 min at 60 °C at Thermomixer Comfort (Eppendorf, Germany). Dialyses for 24 h were performed on membrane filter (0.025 µm VSWP, Millipore) against 2 L of water. This form is designated as “Lip-8-dox”.

### 4.4. Preparation of Myocet, Epirubicin and Doxorubicin

Myocet was obtained from Cephalon (Maisons-Alfort, France) and the solution of liposome was prepared according to the information leaflet. The concentration of doxorubicin was 2 mg/mL. Doxorubicin was purchased from Teva Pharmaceuticals (Pardubice, Czech Republic). Epirubicin was purchased from EBEWE Arzneimittel GmBH (Unterach, Austria).

### 4.5. Cell Cultures

Three human prostatic cell lines were used in this study: (i) PNT1A human cell line established by immortalization of normal adult prostatic epithelial cells by transfection with a plasmid containing the SV40 genome with a defective replication origin. The primary culture was obtained from the prostate of a 35-year old male at post mortem; (ii) 22Rv1 is a human prostate carcinoma epithelial cell line derived from a xenograft that was serially propagated in mice after castration-induced regression and relapse of the parental, androgen-dependent CWR22 xenograft. 22Rv1 cells are androgen-receptor positive, but its levels are lower compared to the LNCaP cell line. 22Rv1 cells harbor wild-type p53. The 22Rv1 cell line is androgen-responsive, yet androgens are not required for growth, although the cell line shows growth response in their presence [35]; (iii) The LNCaP cells line was derived from human prostate adenocarcinoma cells of a 50-year old Caucasian male in 1977, where cells were taken from a needle aspiration biopsy of a metastatic lesion in the left supraclavicular lymph node. Compared to that in the 22Rv1 cell line, the androgen receptor of the LNCaP cell line is well expressed, cells respond highly to androgens–androgens stimulates cell growth [36]. LNCaP is a wild type p53-expressing cell line [35,37]. All cell lines used in this study were purchased from Health Protection Agency Culture Collections (Salisbury, UK).

### 4.6. Culture Conditions

PNT1A, LNCaP, and 22Rv1 cells were cultured in RPMI-1640 medium with 10% FBS. All media were supplemented with penicillin (100 U/mL) and streptomycin (0.1 mg/mL), and the cells were maintained at 37 °C in a humidified incubator (Sanyo, Japan) with 5% CO_2_.

### 4.7. Treatment of Cells

Once the cells reached 50%–60% confluence, the growth media were replaced by fresh medium for 24 h to synchronize cell growth. The cells were then treated with doxorubicin, epirubicin, Myocet, apodox and lip-8-dox with following concentrations used: 0 (control) and 40 µM in fresh medium for 48 h.

### 4.8. Measurements of Cell Viability—MTT Test

The suspension of 5000 cells was added to each well of standard microtiter plates. Volume of 200 μL was transferred to 2–11 wells. Medium (200 μL) was added to the first and to the last column (1 and 12, control). Plates were incubated for 2 days at 37 °C to ensure cell growth. Medium was removed from columns 2–11. Columns 3–10 were filled with 200 μL of medium containing increasing concentrations of cytostatics (0–40 μM). As control, columns 2 and 11 were filled with fresh medium without cytostatics. Plates were incubated for 24; then, media were removed and replaced by a fresh medium, three times a day. Columns 1 to 11 were filled with 200 μL of medium containing 50 μL of MTT (5 mg/mL in PBS) and incubated in a humidified atmosphere for 4 h at 37 °C, wrapped in aluminium foil. After the incubation, MTT-containing medium was replaced by 200 μL of 99.9% dimethyl sulphoxide (DMSO) to dissolve MTT-formazan crystals. Then, 25 μL of glycine buffer was added to all wells and absorbance was immediately determined at 570 nm (VersaMax microplate reader, Molecular Devices, Sunnyvale, CA, USA). All measurements were performed as tetraplicates. A macro programmed in Excel (Microsoft Corp., Redmond, WA, USA) was used for the calculation of half-maximal inhibition concentration (IC_50_). Data was fitted with a logistic function to create sigmoidal dose-response curve. The curve is described by four variables: upper limit, lower limit, skewness of the function and log IC_50_. The fitting was adjusted using an automated algorithm employing a solver function with least squares method. This procedure was used for each repetition and thus variance was calculated from these measurements.

### 4.9. Cell Proliferation Measurement Using Real-Time Cell-Based Assay

The real-time cell-based assay (RTCA) xCELLigence (Roche Applied Science and ACEA Biosciences, San Diego, CA, USA) consists of four main components: the RTCA analyzer, the RTCA station, the RTCA computer with integrated software and disposable E-plate 16. Firstly, the optimal seeding concentration for proliferation and cytotoxic assay was determined. After seeding of the total number of cells in 200 μL medium to each well in E-plate 16, the attachment, proliferation and spreading of the cells was monitored every 15 min. All experiments were carried out for 250 h. The results are expressed as relative impedance using the manufacturer’s software (Roche Applied Science and ACEA Biosciences). Half-maximal concentration was calculated in the RTCA proprietary software using a sigmoidal dose-response formula and by fitting an area under curve in a time period *vs.* concentration curve type. The precision was derived from multiplicative measurement of each concentration.

### 4.10. Determination of Viable Cell Numbers by Propidium Iodide Exclusion

All cells in the culture were harvested and briefly trypsinized and resuspended in PBS. Immediately before measurement, propidium iodide was added to a final concentration of 1 µg/mL, and the number of viable cells was measured by FACSVerse flow cytometer (BD Biosciences, San Jose, CA, USA). The data obtained were analyzed using FACSuite software (BD Biosciences).

### 4.11. Determination of Liposomal Particle Size

The average size of the nanoparticles and the size distribution were determined by quasielastic laser light scattering with a Malvern Zetasizer (NANO-ZS, Malvern Instruments Ltd., Worcestershire, UK). 1.0 mL of water solution of liposome was put into a polystyrene latex cell and measured at a detector angle of 173°, a wavelength of 633 nm, a refractive index of 0.30, a real refractive index of 1.59, and a temperature of 25 °C.

### 4.12. Determination of Cholesterol

Content of cholesterol was determined by absorbance measurement at 505 nm using reagent R1 (Greiner, Frickenhausen, Germany). The measurements are based on our previous experiments [38].

### 4.13. Spectrophotometric Measurements

Fluorescence and absorption spectra were acquired by multifunctional microplate reader Tecan Infinite 200 PRO (Tecan, Männedorf, Switzerland). The excitation wavelength used was 490 nm and the fluorescence scan was measured within the range from 530 to 850 nm per 2-nm steps. The detector gain was set to 90. The samples were placed in a UV-transparent 96-well microplate with a flat bottom by CoStar (Corning, New York, NY, USA). To each well 100 μL of sample was placed. All measurements were performed at 30 °C controlled by Tecan Infinite 200 PRO (Tecan, Männedorf, Switzerland).

### 4.14. Scanning Electron Microscope (SEM)

Structure of liposomes were characterised by an electron microscope (SEM). For documentation of the selected nanomaterials, a FEG-SEM MIRA XMU instrument (Tescan, a.s, Brno, Czech Republic) was used.

### 4.15. Difference Pulse Voltammetry

The three-electrode system consisted of glassy carbon working electrode, an Ag/AgCl/3 M KCl reference electrode and a platinum electrode acting as the auxiliary was used. Settings of the potentiostat were as it follows: scan from −0.3 to −0.9 V using an amplitude of 25 mV, step of 2 mV and frequency of 25 Hz; equilibration time 20 s; nitrogen bubbling during the experiment; cleaning at −1.0 V for 40 s; and stirring during cleaning step.

### 4.16. Statistical Analysis

The half-maximal inhibition concentrations (IC_50_) of individual cytostatics from MTT tests were calculated from sigmoidal dose-response-fitted data. Half-maximal concentration from impedance-based measurement was calculated in software supplied by manufacturer (RTCA 1.2.1). Data was tested for normality using distribution fitting and chi-squared test. Factorial ANOVA was used to compare the effect of individual factors—cell line, cytostatic, and method used (MTT- and impedance-based). When significant difference was determined, Bonferroni *post hoc* test was used. *p*-level < 0.05 was considered significant. Software Statistica (StatSoft, Tulsa, OK, USA) was used for analyses.

## 5. Conclusions

This study focused on comparison of toxicity of novel modified forms of doxorubicin prepared in our lab. Despite the differences between assays, doxorubicin was the most toxic of tested compounds. This study confirmed lower toxicity of commercially available Myocet and also of modifications prepared in our lab—apoferritin-coated apodox and liposome-coated lip-8-dox, which complies with the initial premise. Most importantly, it was demonstrated, that liposomal modification “lip-8-dox” exhibits higher toxicity to tumorous cell lines, but is significantly less toxic for non-tumour cells. Based on this pilot study, precise mechanisms of coated doxorubicin modifications and their effects on *in vivo* models will be the subject of future experiments.
